# Supplementary material for: Quantum-assisted distortion-free audio signal sensing
Source: Nat Commun. 2022 Aug 8;13:4637. doi: 10.1038/s41467-022-32150-1 (PMC9360047; doi:10.1038/s41467-022-32150-1)
Supplement: Supplementary file 1 — Supplementary Information for [file 41467_2022_32150_MOESM1_ESM.pdf]

Supplementary Information: Quantum-assisted distortion-free audio signal sensing

Chen Zhang,[1,*] Durga Dasari,[1,†] Matthias Widmann,[1] Jonas Meinel,[1] Vadim Vorobyov,[1] Polina Kapitanova,[2] Elizaveta Nenasheva,[3] Kazuo Nakamura,[4] Hitoshi Sumiya,[5] Shinobu Onoda,[6] Junichi Isoya,[7] Jörg Wrachtrup[1]

*Corresponding author. Email: chen.zhang@pi3.uni-stuttgart.de; d.dasari@pi3.uni-stuttgart.de;

Here we show (1) Comparison of sensing schemes for distortion-free sensing applications, (2) Comparison of sensors for detecting oscillating fields, (3) the supplemental derivation of the rotating frame modulation, (4) the supplemental derivation of the heterodyne readout, (5) the derivation of the shot-noise limited sensitivity and the estimation of the experimental sensitivity limit, (6) the supplemental information on the frequency resolution, (7) the description of the audio signal sensing details, (8) the algorithm of QPSD spectrum analysis. In addition, audio files related to this work are provided separately.

Supplementary Note 1: Sensing schemes for distortion-free sensing applications

In this note we compare the performances of different measurement schemes for extending dynamic range so that one can have an overview of the advances in technique (see Supplementary Table 1). As discussed in the introduction, the dynamic range of traditional interferometry schemes, e.g., Ramsey, Hahn-echo (HE), dynamical decoupling (DD) sequences, is limited by the sinusoid response, which obtains a small LDR according to the small angle approximation. Here we summarize three schemes that can be used to extend the LDR, i.e., resonance tracking, phase-estimation-algorithm (PEA), and the QPSD method.

The resonance tracking scheme uses feedback to lock the resonance frequency so that the sensor always works in the range that satisfies the linearity requirement. There are two different ways to achieve the resonance tracking measurement. One is to use coils to compensate the changing of the external field. This method is used for integrated OPM sensors [1]. High precision current source is needed to operate the compensation coils in a low noise level. Since the noise level of a current source is usually proportional to the output current, the dynamic range of such a sensor usually comes with the degrading of sensitivity. On the other hand, this regime adds difficulty in using the sensors in array due to the crosstalk over the compensation coils of the sensors [2]. The other regime of the feedback scheme is to shift the frequency of the driving field which is used to resonantly drive the transitions. We take the NV magnetometer as an example. When the frequency of the applied MW fields can track the resonance line shift that induced by changing of the external field, the readout of the sensor also stays in the linear range. The regime can be easily applied to the ODMR measurement [3]. However, there are still technical challenges when the frequency-locking regime is applied to the interferometric methods. Due to the principle that the schemes use the spin-field interaction that response to the amplitude and phase of the oscillating field in a synthesized way, it is not possible to determine the feedback without pre-knowledge of the detected signal. DC field can be measured by Ramsey sequence with a feedback

of the MW frequency shifting, but it is difficult to maintain the strength of the driving field at the same level at different MW frequencies. This leads to imprecise manipulation of the spin states and results to error in the measurements.

In order to extend the dynamic range for the interferometric measurements, the PEA readout scheme is proposed for both dc and ac sensing [4-6]. The scheme estimates the quantum phase constant by using multiple readout of DD sequences, e.g. Carr-Purcell (CP) – 2n sequences, and the field sensing time ranges from short to long with the order of each DD sequence. The sensitivity of this scheme scales with resources used in the measurement, i.e., number of the applied sequences, and the experimental sensitivity value is between the Hahn-echo and the highest order of DD sequence [5]. The detectable dynamic range of measurements that use PEA has no fundamental limit in principle. However, the scheme shows more advantages in estimating the phase of a near static oscillating signal than measuring phase dynamics of a varying signal with the concerning of precision. When the signal varies not in a static way, one has to use less resources for the phase estimation to save bandwidth, which will degrade the sensitivity. Besides, the frequency of the signal to be detected is locked on by the applied sequences. Sequences always need to be adjusted for resolving signals at a different frequency. The frequency resolution is affected by the linewidths of filter functions defined by the applied sequences. It is difficult to combine PEA with heterodyne readout due to the unequal time interval between the fluoresce readout.

The QPSD scheme described in this work shows the feasibility for distortion-free sensing applications. The scheme is behind the idea of lock-in detection, through which the quantum phase sensitive detection is achieved. The sensor readout obtains linearity over the entire phase range of $[-\pi, \pi]$. For magnetic field readout, the dynamic range can be further extended if the reference phase in the demodulation adaptively adjusted to avoid wrapping of the phase. On the other hand, the QPSD scheme is combined with the frequency-offset heterodyne readout, in which the applied sequence does not change while signals around the frequency defined by the sequence can be resolved with a resolution defined by the measurement time. The sensitivity of the QPSD scheme deteriorates with a factor of $\sqrt{2}$ from the sensitivity of the traditional interferometry measurement. Unlike schemes that use PEA of which the sensitivity is degraded due to the low-order DD contribution in the measurement, the QPSD scheme can relies on high-order DD sequence only to acquire a high sensitivity.

Supplementary Table 1. Comparison of schemes for distortion-free ac sensing applications

Schemes	Seq.	Sensitivity limit [a]	Dynamic range	Freq. Res. [b]
Traditional interferometry	HE/DD	$\sim\sqrt{1/T_\phi}$	Small angle approx.	Sequence filter function depends
Q-dyne	HE/DD	$\sim\sqrt{1/T_\phi}$	Small angle approx.	$t_{meas}^{-1.5}$
Feedback	ODMR/Ramsey	$\sim\sqrt{1/T_2^*}$	Hardware limits [c]	Hardware depends for low frequency; Not applicable for high frequency
PEA	HE+CP2+ CP4+…+CP(2^k)	$\sqrt{\frac{1}{(2^k t_{min})}}\sim\sqrt{\frac{1}{t_{min}}}$	No limit for near static signal	Sequence filter function depends
QPSD	HE/DD	$\sim\sqrt{2/T_\phi}$	$[-\pi,\pi]$ or no limit with elimination of phase wrapping	$t_{meas}^{-1.5}$

[a.] This column gives the scaling of the sensitivity limit. T_ϕ is the total interaction time in the applied sequence. t_{min} is the shortest interaction time in the PEA sequence, i.e. interaction time of the HE sequence.
[b.] t_{meas} is the total measurement time. The PEA can hardly utilize heterodyne readout due to the unequal time interval of the fluorescence readout.
[c.] The feedback scheme requires either field compensation or driving field frequency shift. Both of the choices meet challenges, see in Supplementary Note 1.

Supplementary Note 2: Sensors for detecting oscillating fields

To have an overview of the existing techniques that can be used for detecting oscillating fields, we present Supplementary Table 2 as a summary of the specifications of the techniques. The techniques can be used for applications such as telecommunication.

Conventional antennas can be used for detection of both electric and magnetic components of the propagating electromagnetic signals. Both electric and magnetic measurements with antennas obtain very high sensitivity. By designing different types of antennas, the measurements can cover the entire radio spectrum. Nevertheless, antenna techniques meet limitations in challenging environment. Since electromagnetic field attenuated exponentially with the skin depth when propagating through materials e.g. rock and water, conventional wireless communication techniques are hardly to be applied for systems underwater, underground, or inside buildings [7]. The only option is to use low-frequency (LF) and even very-low-frequency (VLF) signals which are less attenuated. However, to be on resonant to the VLF and LF signals, the dimension of the antennas ranges from a meter to hundreds of meters. It could be difficult to install such antennas in applications of which space is limited, e.g. in unmanned underwater vehicles, in underground establishments. In Supplementary Table 2, we present the specifications of the "Atmospheric Weather Electromagnetic System for Observation Modeling and Education" (AWESOME) as an example to compare with the other techniques [8,9].

The recent progress of quantum sensors based on Rydberg atoms shows the potential of the techniques in detect local electric field with a broad bandwidth ranging from dc to 20 GHz [10,11]. The readout of the quantum sensor can surpass the thermal noise limits in conventional electronic receivers. However, the instinct sensitivity of the up-to-date Rydberg sensors is far behind the conventional receivers with antennas. By using waveguide and preamplifier to couple RF signals from antennas to the atomic vapor, the system can make advantage from the antenna gain as conventional receivers. On the other hand, the Rydberg sensors obtain the best sensitivity at the frequency around 10 GHz [11]. For detecting low frequency signals, the sensitivity is deteriorated, and there is the same problem of large antenna dimension as conventional receivers.

Advances of new types of magnetometers path ways for possibilities in wireless communication by detecting magnetic field with high sensitivity. Magnetometers based on vapor cells can achieve sensitivities ranging from $fT/Hz^{1/2}$ to $pT/Hz^{1/2}$. For operating in an unshielded environment, optically pumped magnetometers (OPM) with 0.2 $pT/Hz^{1/2}$ sensitivity is available on the market [12]. However, the OPM has limited bandwidth only from dc to 1 kHz. On the other hand, magnetometers based on NV centers in diamond can measure oscillating fields from VLF band to high-frequency (HF, 3 MHz – 30 MHz) band. The up boundary of the bandwidth depends on the instrumentations and can be higher. Although the reported instinct sensitivity is still at $pT/Hz^{1/2}$ level, there are abundant of techniques, e.g. various dynamical decoupling methods, to be applied for further improvement.

To further improve the detected SNR of the magnetometry system, flux concentrator is proposed to be used as the "antenna" for magnetic flux, that can amplify the local magnetic signal [13,14]. The "antenna gain" of the flux concentrator mainly depends on the ferrite material and the geometry rather than the signal frequency. With a simple estimation from geometry, a ferrite cone that concentrates flux from centimeter to millimeter dimension can improve the signal amplitude by hundreds of times. Since diamonds in NV magnetometers are usually smaller than vapor cells in OPMs, the flux gain can compensate the sensitivity difference between NV magnetometers and the OPMs.

Supplementary Table 2. Summary of sensors for detecting oscillating fields

Type	Sensitivity 1	Sensitivity 2 (dBm/Hz)*	Sensor outline (m)	BW(Hz)	Notes
Antenna [a]	5×10^{-9} (V/m)/Hz$^{1/2}$ [8]	-263 @>20 kHz	2	0.5-470k	"AWESOME" sys. Different antennas available
Rydb. Sensor [b]	3×10^{-3} (V/m)/Hz$^{1/2}$ [10]	-134 @14 GHz	10^{-1}	$0\text{-}10^{10}$	Waveguide, antenna, and amplifier are used and get a sensitivity of -145 dBm/Hz [11]
Antenna [c]	0.03 fT/Hz$^{1/2}$ [9]	-244 @>20 kHz	2.6(L) ×1.3(H)	0.5-470k	"AWESOME" sys. Different antennas available
Search Coils [d]	4 fT/Hz$^{1/2}$ [15]	-202 @6 kHz	0.17	$1\text{-}10^{5}$	Sensitivity deteriorates to pT near DC
OPM [e]	0.2 pT/Hz$^{1/2}$ [12]	-168 @<1 kHz	3.6×10^{-2}	$0\text{-}10^{3}$	Flux concentrator is used for a gain of 19 folds (~25 dB) in SNR [14]
NV [f]	9 pT/Hz$^{1/2}$ [16]	-135 @20 kHz	5×10^{-4}	$0\text{-}10^{6}$	Flux concentrator is used for a gain of 250 folds (~48 dB) in SNR [13]

* Sensitivity 1 is the reported values. We calculated the corresponding noise energy density level in Sensitivity 2 for a direct comparison.

a. The electric sensor of "AWESOME" system uses dipole antennas. The outline is the antenna size for the reported sensitivity obtained at frequencies larger than 20 kHz.

b. The outline refers to the length of the vapor cell used in the system.

c. The magnetic sensor of "AWESOME" system uses a right-isosceles loop antenna. The outline is the antenna size. The best sensitivity is obtained from 20 kHz to 60 kHz.

d. The length of the core for the coils is used as description of the outline. The sensitivity is <10 fT/Hz$^{1/2}$ at frequency band from 1 kHz to 30 kHz.

e. The dimension of the sensor head from twinleaf.com/scalar/OMG/ is used to represent the outline. The sensor has a cross-section of 1.6 mm × 3.6 mm.

f. The value of the sensor outline given in the table is the dimension of the diamond. The reported integrated NV magnetometer includes endoscope in millimeter size and sensor head in centimeter size. The reported best sensitivity in Ref. [16] has been corrected, but still has room for improvement. We note that the sensitivity value presented here does not stand for the best sensitivity over the entire bandwidth.

Supplementary Note 3: Derivation of the rotating frame modulation

Based on Eq. (2) in the main text, the evolution operator with the π/2 pulse duration $\tau_1=\pi/(2\Omega_1)$ can be calculated as

$$e^{-i\frac{\mathcal{H}_1'}{\hbar}t}=\frac{1}{\sqrt{2}}\begin{pmatrix}1 & -ie^{-i(\delta\omega_1\tau_1+\alpha)}\\ -ie^{i(\delta\omega_1\tau_1+\alpha)} & 1\end{pmatrix}. \tag{S1}$$

In the sensing duration, the spin state acquires an extra phase factor due to the external field and can be expressed as

$$\left|\psi(T_\phi)\right\rangle=\frac{1}{\sqrt{2}}\begin{pmatrix}-ie^{-i(\delta\omega_1\tau_1+\alpha+\phi)}\\ 1\end{pmatrix}. \tag{S2}$$

The evolution operator during the MW2 pulse is similar to (S1), and the spin state after the second π/2 pulse is

$$\left|\psi_{meas}\right\rangle=e^{-i\frac{H_2'}{\hbar}t}\left|\psi(T_\phi)\right\rangle=\frac{1}{\sqrt{2}}\begin{pmatrix}e^{-i\Phi}-ie^{-i\delta\omega_2\tau_2+\beta)}\\ -ie^{-i\Phi}e^{i(\delta\omega_2\tau_2+\beta)}+1\end{pmatrix}, \tag{S3}$$

where $\Phi=\delta\omega_1\tau_1+\alpha+\phi+\pi/2$, $\tau_2=\pi/(2\Omega_2)$ is the π/2 duration of MW2. Then, we can calculate the measured expectation value as

$$\left\langle S_z\right\rangle=\frac{\hbar}{2}\left\langle\psi_{meas}\right|\sigma_z\left|\psi_{meas}\right\rangle=\sin\left[\phi+\frac{\pi}{2}\left(\frac{\delta\omega_1}{\Omega_1}-\frac{\delta\omega_2}{\Omega_2}\right)+\alpha-\beta\right], \tag{S4}$$

where $\alpha-\beta=\delta\omega\cdot t$, $\delta\omega=\delta\omega_1-\delta\omega_2=2\pi\delta f$.

Supplementary Note 4: Derivation of frequency offset heterodyne readout

Here we give a general switching function based on the CPMG-n sequence. Typically, a microwave sequence consisting of π-pulses can be described by a switching function

$$g(t)=\begin{cases} 1, & t\in\left[0,\dfrac{T_\phi}{2n}\right) \\ (-1)^k, & t\in\left[\dfrac{2k-1}{2n}T_\phi,\dfrac{2k+1}{2n}T_\phi\right) \\ (-1)^n, & t\in\left[\dfrac{2n-1}{2n}T_\phi,T_\phi\right) \\ 0, & t\in\left[T_\phi,mT_\phi\right] \end{cases}, \tag{S5}$$

where $k\leq n$-1, and k, n, $m\in\mathbb{N}$. The switching function is repeated in each time interval of $[NmT_\phi, (N+1)mT_\phi]$, where $N\in\mathbb{Z}$. Given a random sinusoid signal $B_{ac}(t) = B\mathrm{e}^{-i(\omega t+\varphi)}$, the readout of each cycle is given by $\phi_r(t)=\int_{-\infty}^{\infty}s(t)g(t)dt$. For each sampling point, the accumulated phase factor is

$$\phi_r(N)=\begin{cases} \dfrac{4\left(\sin\dfrac{\omega T_\phi}{4n}\right)^2\left(\cos\dfrac{\omega T_\phi}{2}\right)}{\omega\cos\dfrac{\omega T_\phi}{2n}}e^{i(\frac{\omega T_\phi}{2}-\frac{\pi}{2})}Be^{-i\varphi}e^{-i\omega NmT_\phi}, & n\ is\ odd; \\ \\ \dfrac{4\left(\sin\dfrac{\omega T_\phi}{4n}\right)^2\left(\sin\dfrac{\omega T_\phi}{2}\right)}{\omega\cos\dfrac{\omega T_\phi}{2n}}e^{i(\frac{\omega T_\phi}{2}-\pi)}Be^{-i\varphi}e^{-i\omega NmT_\phi}, & n\ is\ even. \end{cases} \tag{S6}$$

The equation is simplified as

$$\phi_r(N)=\left|G_n(\omega)\right|e^{i\left(-\frac{\omega T_\phi}{2}-\frac{P}{2}\pi\right)}\gamma_e B(\omega)e^{-i\varphi(\omega)}e^{-i\omega NmT_\phi}, \tag{S7}$$

where $G_n(\omega)=\left|G_n(\omega)\right|e^{i\left(-\omega T_\phi/2-P\pi/2\right)}$ is the MW filter function of the sequence. When n=1 and 2, i.e., Hahn-echo and CPMG-2 sequence, the sensor responses are

$$G_1(\omega)=\frac{4}{\omega}\left(\sin\frac{\omega T_\phi}{4}\right)^2 e^{-i\left(\frac{\omega T_\phi}{2}-\frac{\pi}{2}\right)} \tag{S8}$$

$$G_2(\omega)=\frac{8}{\omega}\left(\sin\frac{\omega T_\phi}{8}\right)^2\left(\sin\frac{\omega T_\phi}{4}\right)e^{-i\left(\frac{\omega T_\phi}{2}-\pi\right)} \tag{S9}$$

When $\omega=2\pi/T_\phi$, the two sequences have the equal response as $|G_1| = |G_2| = 2T_\phi/\pi$. The phase response difference is $\pi/2$. Therefore, the Hahn-echo sequence can be used to measure the $X(\omega, t)$ component for the quantum-LIA, in correspondence to a classical LIA. The CPMG-2 sequence can be used to measure the $Y(\omega, t)$ component. Furthermore, we can get $|G_1(2\pi/T_\phi)| = |G_1(\pi/T_\phi)|$ from (S8).

Supplementary Note 5: Derivation of shot-noise limited sensitivity

According to the schematic described in the main text, we try to extract the phase factor from the N samples in a modulation cycle referencing to [17] and 3.4 in [18]. The shot-noise contribution to each sample is $\delta\mathcal{F} = 2\sqrt{\mathcal{N}}$, where $\mathcal{N}$ is the detected photon number in each measurement. The photon counts can be expressed as $\mathcal{N} = \epsilon N_{NV} n_{avg}$, where ϵ is the fluorescence collection efficiency, N_{NV} is the number of involved NV centers, and n_{avg} is the average number of emitted photons per NV center. There is a factor of $\sqrt{2}$ because each sample includes two steps measurement, and another factor of $\sqrt{2}$ comes from the laser reference. The output from the photodetector can be described as

$$s_k = \mathcal{N}C\sin\left(\frac{2\pi k}{N}+\phi\right), \tag{S10}$$

where k denotes the number in the samples and N is the total sample number. Here we use C to represent the detected contrast instead of using $Ce^{-(T_\phi/T_c)^p}$ for simplicity. Since the photodetector output includes the shot-noise, the measured $\overline{s_k}$ include the noise $\delta\overline{s_k} \approx \delta\mathcal{F}$. We can define a minimum error square as

$$\chi^2 = \sum_{k=1}^{N}(\overline{s_k} - s_k)^2. \tag{S11}$$

Additionally, we have $\sum_{k=1}^{N}\sin^2(2k\pi/N+\phi) = N/2$, and $\sum_{k=1}^{N}\sin(2k\pi/N+\phi)\cos(2k\pi/N+\phi) = 0$ when each modulation cycle is equally sampled with an even number. Let $\partial\chi^2/\partial\phi = 0$ to minimize χ^2,

$$\sum_{k=1}^{N}\overline{s_k}\cos\left(\frac{2k\pi}{N}+\phi\right) = 0. \tag{S12}$$

Take a differential,

$$\sum_{k=1}^{N}\left[\delta\overline{s_k}\cos\left(\frac{2k\pi}{N}+\phi\right) - \overline{s_k}\sin\left(\frac{2k\pi}{N}+\phi\right)\delta\phi\right] = 0. \tag{S13}$$

Sample $\overline{s_k}$ can be replaced by $s_k + \delta\overline{s_k}$. We can neglect the high order error $\delta\overline{s_k}\delta\phi$ and get

$$\sum_{k=1}^{N}\delta\overline{s_k}\cos\left(\frac{2k\pi}{N}+\phi\right) \approx \delta\phi\sum_{k=1}^{N}\mathcal{N}C\sin^2\left(\frac{2k\pi}{N}+\phi\right) = \mathcal{N}C\frac{N}{2}\delta\phi. \tag{S14}$$

The statistical average of the phase variances square is

$$\left\langle(\delta\phi)^2\right\rangle = \frac{4}{N^2}\frac{1}{(\mathcal{N}C)^2}\sum_{k=1}^{N}\sum_{l=1}^{N}\left\langle\delta\overline{s_k}\delta\overline{s_l}\right\rangle\cos\left(\frac{2k\pi}{N}+\phi\right)\cos\left(\frac{2l\pi}{N}+\phi\right). \tag{S15}$$

The measurement errors should be uncorrelated, satisfying $\left\langle\delta\overline{s_k}\delta\overline{s_l}\right\rangle = 0$. Therefore,

$$\left\langle(\delta\phi)^2\right\rangle = \frac{4}{N^2}\frac{1}{(\mathcal{N}C)^2}\sum_{k=1}^{N}\left\langle(\delta\overline{s_k})^2\right\rangle\cos^2\left(\frac{2k\pi}{N}+\phi\right) = \frac{2}{N}\frac{1}{(\mathcal{N}C)^2}\delta\mathcal{F}^2. \tag{S16}$$

The short-noise determined phase noise in the modulation cycle $2NT_{seq}$ is

$$\delta\phi = \sqrt{\frac{2}{N}}\frac{\delta\mathcal{F}}{\mathcal{N}C} = \frac{2\sqrt{2}}{\sqrt{N}}\frac{1}{C\sqrt{\mathcal{N}}}. \tag{S17}$$

Regardless of the phase terms in (S7), there is

$$\delta B = \frac{\delta\phi}{\gamma_e G(\omega)}, \tag{S18}$$

from which we see the scalar factor $k_{phase} = \delta\phi/\delta B = \gamma_e G(\omega)$. Therefore, the final sensitivity turns out to be

$$\eta = \delta B\sqrt{2NT_{seq}} = \frac{4}{\gamma_e |G(\omega)| Ce^{-(T_\phi/T_c)^p}}\sqrt{\frac{T_{seq}}{\mathcal{N}}}. \tag{S19}$$

We also notice $k_{phase} = 2\gamma_e T_\phi/\pi$, and $k_{phase} \approx 0.08°/\text{nT}$ when $T_\phi = 12.5$ μs. Experimentally, we have $k_{phase} = 0.071°/\text{nT}$. The discrepancy comes from the sequence that the quantum phase changes between the two reference steps.

In order to compare with the sensitivity of the direct fluorescence readout, we can get the minimum detectable phase when the signal satisfy the small-angle approximation as

$$\delta\phi' = \frac{\delta\mathcal{F}}{C\mathcal{N}} = \frac{2}{C\sqrt{\mathcal{N}}}. \tag{S20}$$

Therefore, the sensitivity of Hahn-echo measurement with fluorescence readout will be

$$\eta_{fl} = \frac{2\sqrt{2}}{\gamma_e |G(\omega)| Ce^{-(T_\phi/T_c)^p}}\sqrt{\frac{T_{seq}}{\mathcal{N}}} \tag{S21}$$

The shot-noise limited sensitivity of the QPSD readout deteriorates from the value of the fluorescence readout by a factor of $\sqrt{2}$.

Taking $|G_1(2\pi/T_\phi)| = 2T_\phi/\pi$ into the equation and replace the term $Ce^{-(T_\phi/T_c)^p}$ with the detected signal contrast C_{det}, we can get a magnetic field sensitivity

$$\eta = \frac{2\pi}{\gamma_e C_{det}\sqrt{\mathcal{N}}}\frac{\sqrt{T_{seq}}}{T_\phi}. \tag{S22}$$

Here we take experimental parameters into the equation (S22) to estimate the shot-noise sensitivity limit with Hahn-echo sequence. The detected photon rate is $R = 4.6 \times 10^{15}$ Hz so that the photon number in one measurement with T_{seq}=100 μs is $\mathcal{N} = 4.6 \times 10^{11}$. The detected contrast C_{det} = 0.19%. With γ_e=2π×(28 Hz/nT), we can calculate when T_ϕ=12.5 μs, the shot-noise limited sensitivity using QPSD readout is η=22 pT/$\sqrt{\text{Hz}}$. When T_ϕ=50 μs, we measure C_{det} = 0.15% and the sensitivity is estimated as η=7 pT/$\sqrt{\text{Hz}}$.

Supplementary Note 6: Frequency resolution

The following is the detailed description of the measurements of Fig. 3d that discusses the frequency resolution of the experiment. Firstly, we apply sequences with T_ϕ = 50 μs, 50 μs±4 ns and T_{seq} = 10T_ϕ to measure a signal at 20.005 kHz, denoted as Meas. 1 in the figure. The reference frequency shifts 1.6 Hz due to the changing of 1/T_ϕ. As a result, a heterodyne frequency of 6.6 Hz can be detected by measuring a 20.005 kHz field with T_ϕ=50 μs−4 ns. Correspondingly, a 3.4 Hz signal will be detected when measuring a 19.995 kHz field so that it becomes distinguishable to the 20.005 kHz field. For fields at frequencies near the other references ω_{ref}, the shifts can also be calculated accordingly by $k/(mT_\phi)$. To further understand how sensitive is ω_{ref} to the sequence parameters, we only differ ΔT_{seq}=±4 ns to measure external fields at 20.005 kHz and 16.005 kHz while keeping T_ϕ =50 μs. In this case, the heterodyne signal is introduced only by the accumulated phase error in each cycle. The reference frequency shift is calculated by Eq. (7) in the main text. For example, by measuring a signal around 16 kHz with ΔT_{seq}=4 ns, we detect a heterodyne frequency shift of 128 mHz as shown in Fig.3d. Therefore, the frequency fidelity is majorly limited by the jitter performance of the pulse generator. We calculate an example that has a timing error <3 ps, according to the datasheet of the pulse generator we use (Tektronics, DTG5274). The frequency fidelity is 0.06 mHz when the measured signal is around 10 kHz.

Supplementary Note 7: Distortion-free measurement examples – Audio signal sensing

In Fig. 4(a) and (b) we demonstrate the detection of arbitrary fields. In order to have a straightforward looking at the distortion-free measurements, we make two examples by broadcasting two files that contain two pieces of audio signals for detection. In this note, we show details of how the audio signals are processed, and recovered from the QPSD readout. Then, we show more discussions on the low-frequency telecommunication applications.

The first file is a piece of melody that we composed with tones C5, D5, and E5. The corresponding frequencies are f_1=523 Hz, f_2=587 Hz, f_3=659 Hz. The signal waveform plotted in the time domain can be seen in Supplementary Figure 1. We choose the three tones with their frequency differences within 200 Hz because the used quantum phase modulation frequency is 500 Hz and the LIA limited bandwidth is 200 Hz. The signal is modulated with a carrier of 9.5 kHz so that the transmitted magnetic field is at frequencies around 10 kHz, which can be detected by the sensor using the Hahn-echo sequence with T_ϕ=50 μs. Then, the QPSD readouts of the three tones will be at frequencies f_{r1}=23 Hz, f_{r2}=87 Hz, f_{r3}=159 Hz. In order to reconstruct the original signal, the readout is mixed with a 500 Hz carrier. Three notch filters are applied to the mixed signal to remove the sideband as the final step.

In the second example, we play around with a cut of speech. The signal has more complex dynamics on all the specifications, i.e. amplitudes, frequency and phase, than the melody signal. The signal is distributed over a frequency range from DC to a few kHz. This makes it difficult to use the NV sensor in this work to detect the signal in real time due to the limited measurement bandwidth. However, we can compress the signal in frequency domain and have post-processing on the detected signal. We cut off the spectrum from 4 kHz, shown in Supplementary Figure 2(a), because signal amplitude after 4 kHz is much smaller than the components at lower frequencies. In order to make the signal detectable by the sensor, we compress the signal by using interpolation while keeping the same sampling rate for broadcasting. The signal size after interpolation is 20 times the original signal size so that in the frequency domain, the bandwidth is compressed from 4 kHz to 200 Hz, as shown in Supplementary Figure 2(b). Then, the signal is mixed with a 10 kHz reference so that we can use the same Hahn-echo sequence to measure. Supplementary Figure 2(c) shows the spectrum in which the audio signal spectrum distributes in symmetric to 10 kHz. Finally, the QPSD readout detects the heterodyne signal, of which the frequency is referenced to 10 kHz. To reconstruct the original sound, we compress the readout in time domain, i.e., averaging the readout by every 20 data points. In the frequency domain, the spectrum bandwidth is expanded from 200 Hz back to 4 kHz.

The second demonstration shows the current experimental limit in this work, i.e., the low measurement bandwidth despite the wide detectable frequency range. It is possible to further improve the measurement bandwidth in this setup. For example, we can use a higher MW frequency offset δf to enlarge the possible readout bandwidth with the lock-in amplifier. Essentially we could also use carrier with a higher frequency, e.g. 100 kHz, together with the higher MW frequency offset e.g. $\delta f = 5$ kHz to avoid the loss in sampling before the demodulation 2. However, using the higher carrier frequency leads to higher attenuation of electromagnetic signals in water or rocks. Regarding this trade-off in telecommunication system for challenging environment, it is common to compromise with time-lag with the accuracy. Nevertheless, it is still interesting to see the system can receive signals in a more efficient way.

Hereby we outlook the technical possibility that encoding digital signals onto the phase of carriers at different frequencies. The transmitted data rate on each carrier is limited by the measurement bandwidth of the sensor. With the high frequency resolution of the heterodyne detection, there could be a high capacity of the carrier frequencies in the bandwidth. As Fig. 4 (b) shows, phase dynamics at different frequencies within the bandwidth can be detected through the heterodyne detection.

Supplementary Audio 1. (separate file)

'melody.wav' is the original melody signal for detection.

Supplementary Audio 2. (separate file)

'det_melody.wav' is the magnetometer detected melody signal.

Supplementary Audio 3. (separate file)

'voice.wav' is the original voice signal for detection.

Supplementary Audio 4. (separate file)

'det_voice.wav' is the magnetometer detected speech signal.

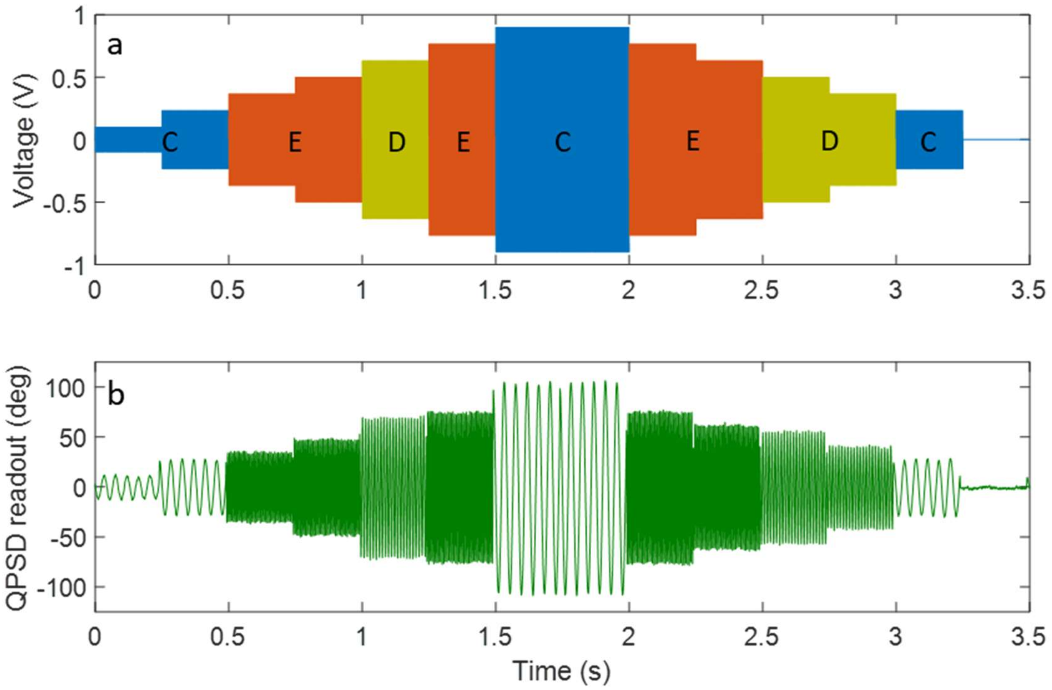

Supplementary Figure 1. (a) Signal waveform of the melody played for the detection. The voltage signal is sent to an AWG for generating the magnetic field through a loop antenna. The different colors represent different scales of the tones that are labeled in the figure. Changing the amplitude indicates that the loudness of the sound rises at the beginning and then falls in the end. **(b)** The QPSD readout of the sensor. Different tones can be recognized from the different heterodyne frequencies in the readout.

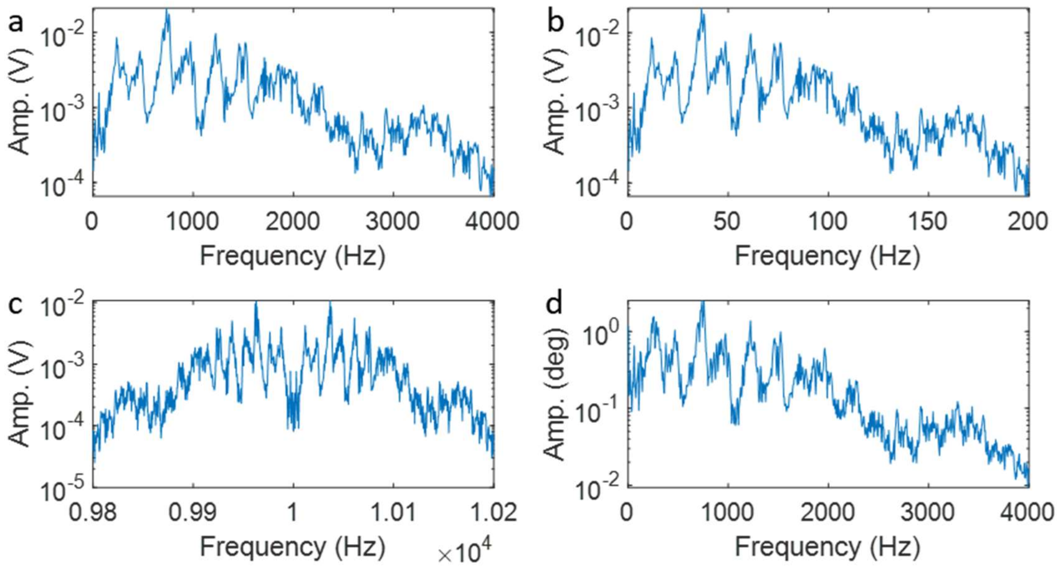

Supplementary Figure 2. (a) Spectrum of the original speech signal. The signal is written into an AWG in the unit of Volt. Therefore, the amplitudes of the spectra in sub-figure A to C are all in Volt. **(b)** Frequency compression. The signal is interpolated to a data size of 20 times larger than the original one. Therefore, the signal is compressed in the frequency domain. **(c)** The signal spectrum after mixing with a 10 kHz reference signal. **(d)** The QPSD readout spectrum. The heterodyne signal is sampled every 20 data from the raw readout to reconstruct the audio signal spectrum.

Supplementary Note 8: Spectrum analysis algorithm

Due to instrumentation in experiments, the QPSD spectrum usually consists of many unexpected noise spikes which are not originated from the magnetic field. We take an example from the measurement of a signal generated around 10.4 kHz. As described in Methods C, $T_\phi = 48\,\mu s$ is applied in the first measurement, and $T'_\phi = 48\,\mu s + 20\,ns$ is applied for the second measurement. By using the measurement sequences with $T_{seq} = 2T_\phi$, the targeted center frequencies of the two measurements are f_{c1}=10.41667 kHz and f_{c2}=10.41233 kHz. As a result, the detected QPSD heterodyne readout spectrum will have a line shift of Δf =4.34 Hz. In Supplementary Figure 3(a), we plot the QPSD spectra of the two measurements. The peaks at the heterodyne frequencies of 16.66 Hz and 12.32 Hz, which have a 4.34 Hz difference, indicate that a magnetic field signal at $f_{c1} - 16.66$ Hz ≈ 10.4 kHz is detected. However, there are still many spikes in the spectra except for the line of the targeted signal. Some of the spikes can be real magnetic field signals, e.g. spectrum leakage due to the digitized signal generated by the AWG, while the others are non-magnetic noises.

To filter out the non-magnetic noises, we use the golden standard that the detected heterodyne frequency of the magnetic field signal shifts by df in the second measurement. Firstly, we set a threshold to pick out all the spikes that need to be distinguished. Since the folded QPSD spectrum includes signals from both sides of the detecting center frequency determined by the sequence, we add the $\pm\Delta f$ shifts to the frequencies of the collected spikes in the spectrum resolved by the first measurement. Then, the shifted frequencies are searched in the list of the collected frequencies of spikes in the spectrum resolved by the second measurement. If the frequency $f_i - \Delta f$ exists in the second list, the signal spike should be plotted at $f_{c1} - \Delta f$; if $f_i + \Delta f$ exists in the list, the signal spike should be plotted at $f_{c1} + \Delta f$; otherwise, we confirm that the spike is not a magnetic field signal. In Supplementary Figure 3(b), the filtered spectrum is plotted in comparison with the QPSD spectrum acquired in the first measurement. The threshold is set at 0.6° in the calculation. From the results in Supplementary Figure 3(b), we find most of the spikes in the direct QPSD spectrum are actually non-magnetic signals. Except of the targeted spike at -16.66 Hz, there are harmonic magnetic fields detected in a frequency distance of 100 Hz. At low heterodyne frequencies when the noise is higher than the threshold, the algorithm keeps the frequencies of the noise and fails in filtering out the non-magnetic signals. There are also spikes lower than the threshold that cannot be picked up. A threshold that is adaptive to the SNR of the detected spectrum can help to improve the algorithm for a better filtering of the non-magnetic signal. We make a Matlab m-function to execute the algorithm in the manner of post-processing. The m-file will be shared on request.

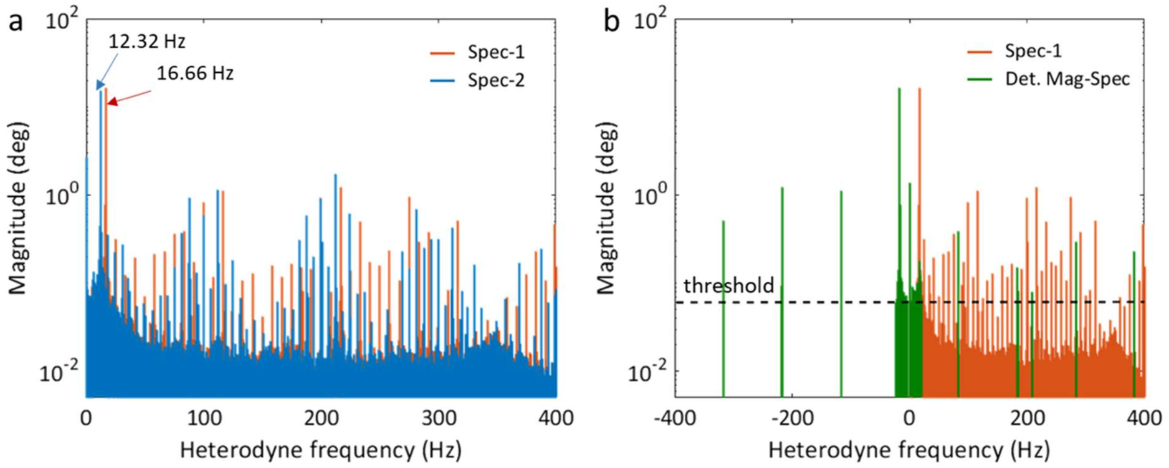

Supplementary Figure 3. (a) The QPSD spectrum measured by sequences using $\boldsymbol{T_{\phi}}$ (Spec-1) and $\boldsymbol{T'_{\phi}}$ (Spec-2). The magnetic field signal is generated by a coil driven by an AWG, with an expected heterodyne frequency at 16.66 Hz. **(b)** Magnetic field spectrum is calculated by using the algorithm that analyzes the heterodyne frequency shift. The threshold is used to recognize signal spikes from the noise. The spectrum is plotted with a comparison to Spec-1.

Reference:

[1] V. K. Shah and R. T. Wakai, *A compact, high performance atomic magnetometer for biomedical applications* Phys Med Biol **58**, 8153 (2013).

[2] N. V. Nardelli, S. P. Krzyzewski, and S. A. Knappe, *Reducing crosstalk in optically-pumped magnetometer arrays* Phys Med Biol **64** (2019).

[3] H. Clevenson, L. M. Pham, C. Teale, K. Johnson, D. Englund, and D. Braje, *Robust high-dynamic-range vector magnetometry with nitrogen-vacancy centers in diamond* Appl Phys Lett **112** (2018).

[4] N. M. Nusran, M. U. Momeen, and M. V. G. Dutt, *High-dynamic-range magnetometry with a single electronic spin in diamond* Nat Nanotechnol **7**, 109 (2012).

[5] N. M. Nusran and M. V. G. Dutt, *Dual-channel lock-in magnetometer with a single spin in diamond* Phys Rev B **88** (2013).

[6] G. Waldherr, J. Beck, P. Neumann, R. S. Said, M. Nitsche, M. L. Markham, D. J. Twitchen, J. Twamley, F. Jelezko, and J. Wrachtrup, *High-dynamic-range magnetometry with a single nuclear spin in diamond* Nat Nanotechnol **7**, 105 (2012).

[7] V. Gerginov, F. C. S. da Silva, and D. Howe, *Prospects for magnetic field communications and location using quantum sensors* Rev Sci Instrum **88** (2017).

[8] B. V. Gurses, K. T. Whitmore, and M. B. Cohen, *Ultra-sensitive broadband "AWESOME" electric field receiver for nanovolt low-frequency signals* Rev Sci Instrum **92** (2021).

[9] M. B. Cohen, R. K. Said, E. W. Paschal, J. C. McCormick, N. C. Gross, L. Thompson, M. Higginson-Rollins, U. S. Inan, and J. Chang, *Broadband longwave radio remote sensing instrumentation* Rev Sci Instrum **89** (2018).

[10] J. A. Sedlacek, A. Schwettmann, H. Kubler, R. Low, T. Pfau, and J. P. Shaffer, *Microwave electrometry with Rydberg atoms in a vapour cell using bright atomic resonances* Nature Physics **8**, 819 (2012).

[11] D. H. Meyer, P. D. Kunz, and K. C. Cox, *Waveguide-Coupled Rydberg Spectrum Analyzer from 0 to 20 GHz* Phys Rev Appl **15** (2021).

[12] A. Jaufenthaler, T. Kornack, V. Lebedev, M. E. Limes, R. Korber, M. Liebl, and D. Baumgarten, *Pulsed Optically Pumped Magnetometers: Addressing Dead Time and Bandwidth for the Unshielded Magnetorelaxometry of Magnetic Nanoparticles* Sensors-Basel **21** (2021).

[13] I. Fescenko, A. Jarmola, I. Savukov, P. Kehayias, J. Smits, J. Damron, N. Ristoff, N. Mosavian, and V. M. Acosta, *Diamond magnetometer enhanced by ferrite flux concentrators* Phys Rev Res **2** (2020).

[14] W. C. Griffith, R. Jimenez-Martinez, V. Shah, S. Knappe, and J. Kitching, *Miniature atomic magnetometer integrated with flux concentrators* Appl Phys Lett **94**, 023502 (2009).

[15] H. C. Seran and P. Fergeau, *An optimized low-frequency three-axis search coil magnetometer for space research* Rev Sci Instrum **76** (2005).

[16] T. Wolf, P. Neumann, K. Nakamura, H. Sumiya, T. Ohshima, J. Isoya, and J. Wrachtrup, *Subpicotesla Diamond Magnetometry* Phys Rev X **5** (2015).

[17] M. Montgomery and D. Odonoghue, *A derivation of the errors for least squares fitting to time series data* Delta Scuti Star Newsletter **13**, 28 (1999).

[18] S. M. Kay, *Fundamentals of statistical signal processing: estimation theory* (Prentice-Hall, Inc., 1993).
