# Supplementary material for: Quantum-assisted distortion-free audio signal sensing
Source: Nat Commun. 2022 Aug 8;13:4637. doi: 10.1038/s41467-022-32150-1 (PMC9360047; doi:10.1038/s41467-022-32150-1)
Supplement: Supplementary file 3 — Description to Additional Supplementary Information [file 41467_2022_32150_MOESM3_ESM.pdf]

Description of Additional Supplementary Files:

Supplementary Audio 1: ‘melody.wav’ is the original melody signal for detection.

Supplementary Audio 2: ‘det_melody.wav’ is the magnetometer detected melody signal.

Supplementary Audio 3: ‘voice.wav’ is the original voice signal for detection.

Supplementary Audio 4: ‘det_voice.wav’ is the magnetometer detected speech signal.
